# Scanning Plasmon-Enhanced Microscopy for Simultaneous Optoelectrical Characterization

**DOI:** 10.1021/acsnano.4c04671

**Published:** 2024-07-27

**Authors:** Joanna Symonowicz, Atif Jan, Han Yan, Manish Chhowalla, Giuliana Di Martino

**Affiliations:** Department of Materials Science and Metallurgy, University of Cambridge, 27 Charles Babbage Road, Cambridge CB3 0FS, United Kingdom

**Keywords:** scanning microscopy, nanoelectrodes, simultaneous characterization, in operando, electrical and optical characterization, plasmonics

## Abstract

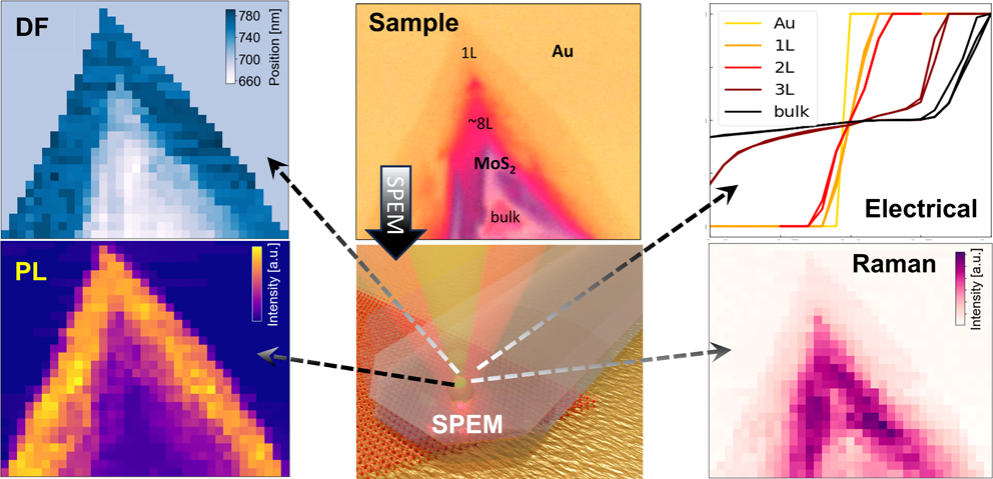

Scanning microscopy methods are crucial for the advancement of nanoelectronics. However, the vertical nanoprobes in such techniques suffer limitations such as the fragility at the tip–sample interface, complex instrumentation, and the lack of in operando functionality in several cases. Here, we introduce scanning plasmon-enhanced microscopy (SPEM) and demonstrate its capabilities on MoS_2_ and WSe_2_ nanosheets. SPEM combines a nanoparticle-on-mirror (NPoM) configuration with a portable conductive cantilever, enabling simultaneous optical and electrical characterization. This distinguishes it from other current techniques that cannot provide both characterizations simultaneously. It offers a competitive optical resolution of 600 nm with local enhancement of optical signal up to 20,000 times. A single gold nanoparticle with a 15 nm radius forms pristine, nondamaging van der Waals contact, which allows observation of unexpected p-type behavior of MoS_2_ at the nanoscale. SPEM reconstructs the NPoM method by eliminating the need for extensive statistical analysis and offering excellent nanoscale mapping resolution of any selected region. It surpasses other scanning techniques in combining precise optical and electrical characterization, interactive simplicity, tip durability, and reproducibility, positioning it as the optimal tool for advancing nanoelectronics.

## 1 Introduction

Despite advancements in nanoelectronics, the challenge to achieve effective nanoelectrodes for research and development of nanomaterials still endures.^1^ The conventional recourse of electron beam lithography^2^ (EBL) proves time-intensive, costly, and often destructive. At the same time, the standard microsized metallic pads limit the detection of local spatial variations, which is crucial for advancing nanoelectronics.^3^

Efforts to realize nanosized electrodes have led to scanning probe methodologies, as detailed in Table 1. Among the techniques are conductive atomic force microscopy (CAFM),^1^ scanning tunneling microscopy (STM),^4^ scanning near-field optical microscopy (SNOM),^5^ scanning capacitance microscopy (SCM),^6^ Kelvin probe force microscopy (KPFM),^7^ scanning thermal microscopy (SThM),^8,9^ scanning photocurrent microscopy (SPCM),^10,11^ scanning microwave impedance microscopy (sMIM),^12,13^ and scanning gate microscopy (SGM).^14,15^

**Table 1:**
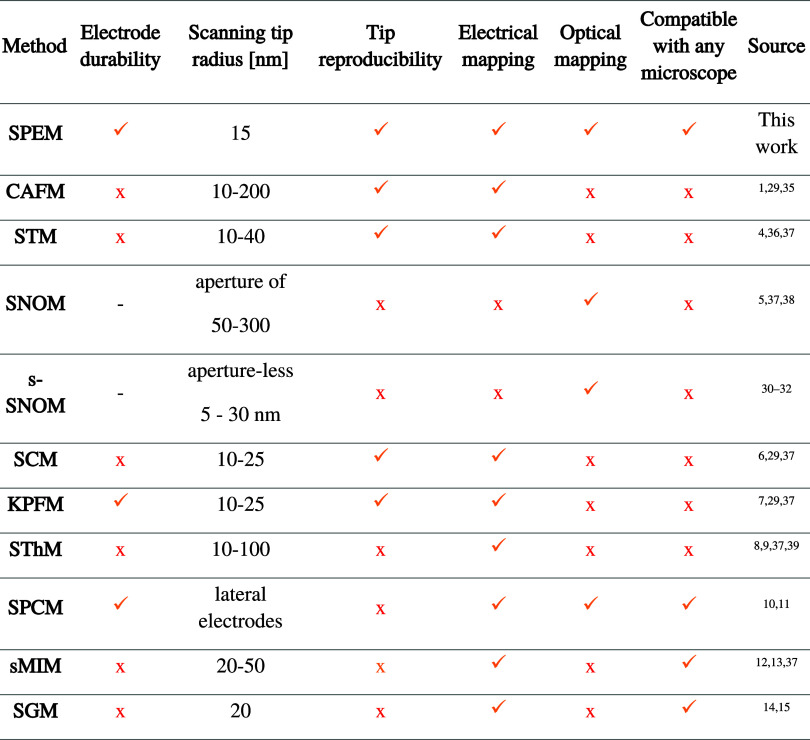
Literature Review of the Standard Scanning Probe Techniques^35−36,37,38,39^

C-AFM, an adaptation of atomic force microscopy (AFM), can image sample surface’s morphology and electrical properties at the microscopic scale,^16^ even in ambient conditions.^17^ The challenge in techniques derived from AFM is the delicacy of tip–sample manipulation, yielding potential damage to both entities and making the estimation of true contact area extremely difficult.^18^ Moreover, these methods employ a specialized setup in which the cantilever position is laser-controlled. In SCM, which measures capacitance changes (∼attofarad), ultrahigh capacitance sensitivity is needed for great spatial resolution. Despite the difficulty of detecting such a small static capacitance, a dynamic change may be measured.^19^ SThM conducts thermal measurements and imaging achieving temperature precision of ∼15 mK and spatial resolution of ∼10 nm in an ultrahigh vacuum (UHV).^20^ In SGM, electrons are scattered by a charged AFM tip-induced potential,^21^ reaching resolution below 10 nm.^22^ KPFM quantifies the contact potential difference between tip and sample,^23^ with a resolution of 50 nm^24^ and even lower 20 nm (i.e., tip size).^25^ SCM, SThM, SGM, and KPFM also derive from the AFM technique, hence sharing the delicacy of tip–sample manipulation and the need for a specialized setup. In STM, variation in tunneling current between tip and surface results in a resolution of 0.01 nm in depth and a resolution of 0.1 nm laterally,^26^ but it necessitates a high-vacuum environment, altering the behavior of materials designed for air operation.^3^ SPCM uses a focused laser beam as an excitation source instead of a tip. Advanced SPCM methods like electron beam-induced current (EBIC) employ electron beams to achieve sub-100 nm to micron precision.^27^ SPCM does not allow for vertical voltage stress. SNOM achieves a resolution of 50–100 nm regularly and may potentially reach 10–30 nm, with aperture SNOM being the most extensively used and highly developed near-field optics technology.^28^ Besides, efforts to electrically bias SNOM tips for concurrent optical and electrical measurements are limited by tip reproducibility.^29^ The best-reported resolution results (down to 5–30 nm, i.e., tip size) were obtained using the apertureless SNOM (s-SNOM).^30,31^ Nonetheless, the main challenge with s-SNOM is the capacity to distinguish light solely from under the tip, compared to a large background signal from the surrounding area.^30,32^ sMIM probes the local tip–sample admittance, susceptible to local sample conductivity, reaching a spatial resolution of ca. 50–100 nm.^33^ Ultrahigh-resolution MIM may attain <5 nm resolution by tip modification, similar to the improvement seen in AFM.^34^ Both sMIM and SNOM require sophisticated and precise instrumentation.

Despite their merits, none stands as a universal solution.^1,3^ Specifically, there is a strong desire for the ability to characterize 2D materials and thin films in their natural environment,^40^ with the capability to simultaneously characterize their topography, electrical properties, and spectroscopic behavior. These materials have a high specific surface area, efficient electron transport channels, and surfaces that can be adjusted and accessed. The methods mentioned in Table 1, including CAFM, STM, STS, SCM, KPFM, SThM, SPCM, SMIM, and SGM, offer topography images, local density of states, conductivity maps, surface potential maps, complex impedance, and other data. However, they do not provide spectroscopic results such as Raman and photoluminescence. On the other hand, tip-enhanced Raman scattering^41^ (TERS), tip-enhanced photoluminescence^42^ (TEPL), second harmonic generation^43^ (SHG), and sum frequency generation^44^ (SFG) techniques offer spectroscopic data, chemical composition analysis, and crystal structure information, among other details. TERS at STM junctions offers superior spatial and optical resolution in comparison to SPEM. However, in contrast to SPEM, STM-TERS apparatuses are intricate and costly. Specifically, the synthesis procedure of TERS tips for achieving single-digit nanometer precision is complex,^45−49^ with tips much more costly compared to SPEM probes.

## 2 Results and Discussion

SPEM, can simultaneously acquire topographic, spectroscopic, and electrical information with plasmon-enhanced nanoscale resolution, while addressing the high demand for user-friendly, simple, and reliable nanoelectrodes.^1,3^ This also enables *in operando* electro-optical characterization of active devices.^50^ In an earlier work,^51^ a similar approach using a diamond tip was introduced for optical imaging; however, it did not allow for electrical characterization. Moreover, SPEM’s far-field scattering is well described and reproducible unlike s-SNOM, and thus, it may be leveraged to obtain insights into the researched material rather than a nuisance background. We demonstrate the excellent capabilities of SPEM on 2D transition metal dichalcogenides (TMDs), such as molybdenum disulfide (MoS_2_) and tungsten diselenide (WSe_2_), due to their sharp layer boundaries, discernible both from microscope imagery and variations in thickness-dependent refractive index (*n*)^52^ as well as intensities of Raman^53^ and photoluminescence (PL)^54^ signals.

### 2.1 Nanoscale Electrical Contact

**Figure 1:**
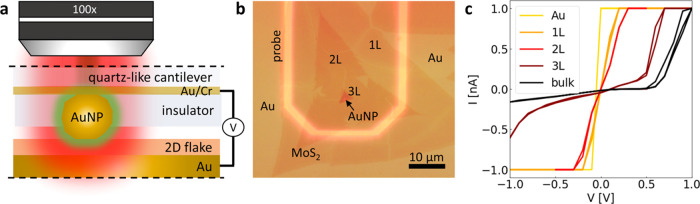
Plasmonic nanoprobes. (a, b) Microscope image (dark-field image overlaid on a bright-field picture). (c) Current–voltage (*I*–*V*) characteristics obtained by SPEM for different thicknesses of the MoS_2_ flakes.

The SPEM is inspired by a well-studied nanoparticle-on-mirror (NPoM) configuration, where a material of interest is positioned between a gold nanoparticle (AuNP) and a plasmonic substrate, typically gold (Au).^50,55−59^ When exposed to light, interactions between electrons in the AuNP and those in the Au substrate create a plasmonic hotspot in the narrow gap between them, thus in the tested material. This hotspot significantly amplifies optical signals.^59,60^ In the case of SPEM, the AuNP can additionally be electrically biased and freely positioned across the sample surface. To achieve this, the AuNP is placed on an optically transparent and electrically conductive cantilever fixed to an XYZR motion stage. An electrical contact is formed through a small Au facet, approximately 30 nm in diameter.^56^ (The size of the AuNP facets has been previously determined in experiments and is well documented, with values typically ranging from 20 to 30 nm in diameter and approximately 15 nm in radius.^56,58,61^ The contact area, therefore, is π*r*^2^ = π(15)^2^ = ∼700 nm^2^.) This is accomplished by coating the entire cantilever area with an insulating polymer, e.g., parylene, except for the top of the AuNP, as depicted in Figures 1a,b and S1.

Utilizing AuNP as a nanoelectrode offers several advantages. It provides nearly miniature vertical electrodes (comparable to SCM, KPFM) available (see Table 1), enabling precise electrical contact, e.g., single 2D materials flakes (Figure 1b—three layers of MoS_2_ of area ≈2 μm^2^), as well as offering pristine van der Waals interface, precise enhanced spectroscopy, and refractive index tracking,^50,55^ as detailed below.

### 2.2 Pristine Interface

Thanks to the lack of adhesion layer (e.g., Ti or Cr) and ligands (removed from the top facet by etching, leaving AuNP stabilized by the parylene coating^55^), SPEM delivers an immaculate van der Waals (vdW) interface. This is confirmed by the current–voltage (*I*–*V*) characteristics for different thicknesses of MoS_2_ (see Figure 1c). Single- and bilayer MoS_2_ present no barrier for tunneling between the Au electrodes. As the MoS_2_ thickness increases, it exhibits p-type semiconductor behavior. This is unexpected as MoS_2_ is widely considered an n-type semiconductor,^60,62^ and p-type behavior is rarely observed.^62^ This suggests that our setup’s electrical contact between the Au electrodes and MoS_2_ is pristine–akin to vdW contacts.^60^ A clean metal/semiconduction junction between MoS_2_ (valence band energy ∼5.75 eV) and Au (work function ∼5.30 eV) results in a relatively small barrier of 0.45 eV for hole injection without an applied voltage.^62^ This contrasts the typical case where the Fermi level of the junction is pinned close to the conduction band of MoS_2_, and therefore only n-type behavior is observed.^62^ The observed p-type behavior suggests that the Fermi level is unpinned and can be modulated by the applied voltage. Thus, our nanoprobe can achieve nanoscale clean vdW contacts, making it a powerful tool for studying the electronic properties of 2D TMDs without the influence of defects at metal/semiconductor junctions.

### 2.3 Simultaneous Enhanced Optical and Electrical Mapping

To the best of our knowledge, simultaneous optical, electrical, and topographical mapping of nanomaterials into a straightforward scanning tool has not been achieved. While alternating between spectroscopic methods requires the change of excitation source, we do it nearly instantaneously using flip mirrors without repositioning the nanoprobe. In the meantime, electrical probing occurs independently, enabling real-time assessment of the influence of vertical bias on the sample’s spectroscopy and, thus, morphology. Notable previous studies^41,42^ show advanced resolution with TERS and TEPL; however, several challenges remain, especially concerning the implementation of ambient environments and in operando studies.^63^

**Figure 2:**
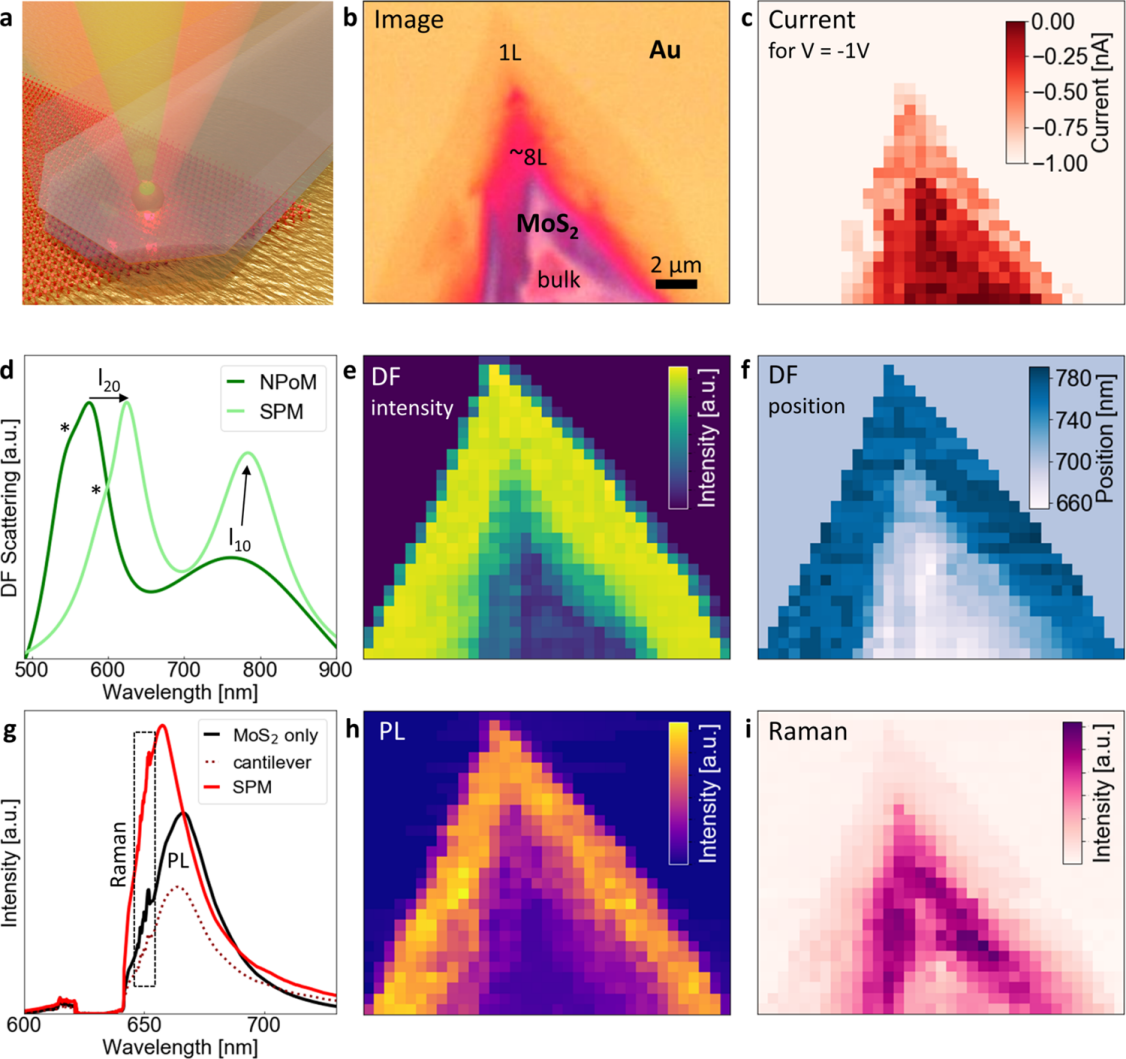
Combined optoelectrical characterization of a MoS_2_ with SPEM. (a) Visualization, (b) microscope image of the mapped MoS_2_ flake, and (c) the corresponding currents measured at *V* = −1 V. (d) Comparison of the plasmonic resonances (single AuNP mode: *, gap dipole: l_10_, gap quadrupole: l_20_) produced by NPoM and SPEM techniques. (e) Intensity and (f) blue shift of dark-field resonant gap mode peak from 780 nm in 1L to 660 nm in bulk MoS_2_. (g) Enhanced Raman and PL spectra of MoS_2_ with SPEM using resonances shown in (d). Intensity maps of the enhanced (h) PL and (i) Raman signals.

To show these combined capabilities, we map MoS_2_ (Figure 2a) displaying thicknesses from monolayer to bulk, as determined from a microscope image (Figure 2b). Figure 2c provides a current map for bias *V* = −1 V, extending Figure 1c, showing consistent shorting for 1–2 layers of MoS_2_ and expected diode-like behavior for the bulk.^60^ Simultaneously with electrical maps, dark-field maps (Figure 2e,f) as well as Raman and PL maps (Figure 2h,i) can be acquired. Unlike AFM-based scanning techniques, SPEM achieves them without requiring extensive data postprocessing, such as calibration, noise subtraction, and data smoothing.

The plasmonic hotspot modes produced by the SPEM can be detected using dark-field (DF) spectroscopy, accomplished by illuminating the AuNP-material-Au configuration with white light (450–950 nm) and detecting resonance wavelengths. As shown in Figure 2d, the plasmonics of SPEM and standard NPoM are comparable.^55^ Notably, in standard NPoMs, the dipolar gap mode l_10_ is damped, most likely by ligands at the AuNP/material interface^55^ (red-shifted modes due to parylene coating—see Figure S2a). This is not observed for SPEM, indicating again a pristine junction that guarantees reliable optical mapping. The DF map of a MoS_2_ flake (Figure 2e,f) demonstrates that with increasing thickness of MoS_2_, the gap resonance, i.e., DF signal, decreases in intensity and blue-shifts (The resonant gap mode undergoes a blue shift, changing its wavelength from 780 nm in 1L MoS_2_ to 660 nm in bulk MoS_2_ referred to as DF position in Figure 2f), consistent with literature data,^56,64,65^ providing this way information about material’s morphology (though not at the highest resolution,^66^ the results are yet sufficiently significant to analyze the morphology of 2D materials and thin films down to grain size ∼15 nm^67−70^), while also providing in operando simultaneous electrical and optical data. In earlier work, a scanning-focused refractive index microscopy^71^ implemented a design to achieve refractive index profiles using focused laser spots; however, it cannot do in operando characterization, and the resolution is limited to 1 μm.

Moreover, the plasmonic modes enhance PL and Raman signals, as presented in Figure 2g. In fact, while the coated cantilever alone reduces the PL (dotted brown line), the presence of AuNP restores and boosts it (red solid line). The enhancement for MoS_2_ is only 2-fold because the plasmonic mode is perpendicular to the gold mirror,^59^ whereas MoS_2_ exciton is parallel.^72,73^ However, for most materials, the perpendicular polarization component would result in SPEM enhancing the Raman spectra by >20,000 times, as demonstrated for PMMA (Figure S3). Notably, with decreasing thickness of MoS_2_, PL gradually fades (Figure 2h) while Raman intensities (Figure 2i) are consistent with established observations.^53,74^ However, for bulk MoS_2_, the Raman signal weakens, which is in opposition to data obtained via standard laser mapping.^53^ This is due to reduced plasmonic enhancement at AuNP-mirror distances exceeding 20 nm,^65^ as visible in Figure 2e.

Another advantage of SPEM is the ability to tune resonances by adjusting coating thickness (see Figure S2b) to match PL/Raman energies from the tested specimen, hence maximizing the enhancement. This is proved by the seeming blue shift in the PL of MoS_2_ upon contact with AuNP (Figure 2g) due to the quadrupolar gap l_20_ resonating at 627 nm, which enhances the bluer portion of the PL spectrum to a greater extent. This aligns with ref (73) and with the statistical analysis presented in Figure S4, where DF resonance wavelengths are tuned by the presence of PMMA to be either greater or smaller than the PL maximum of MoS_2_, which results in red- and blue-shifting of the PL.

### 2.4 Optical Spatial Resolution

**Figure 3:**
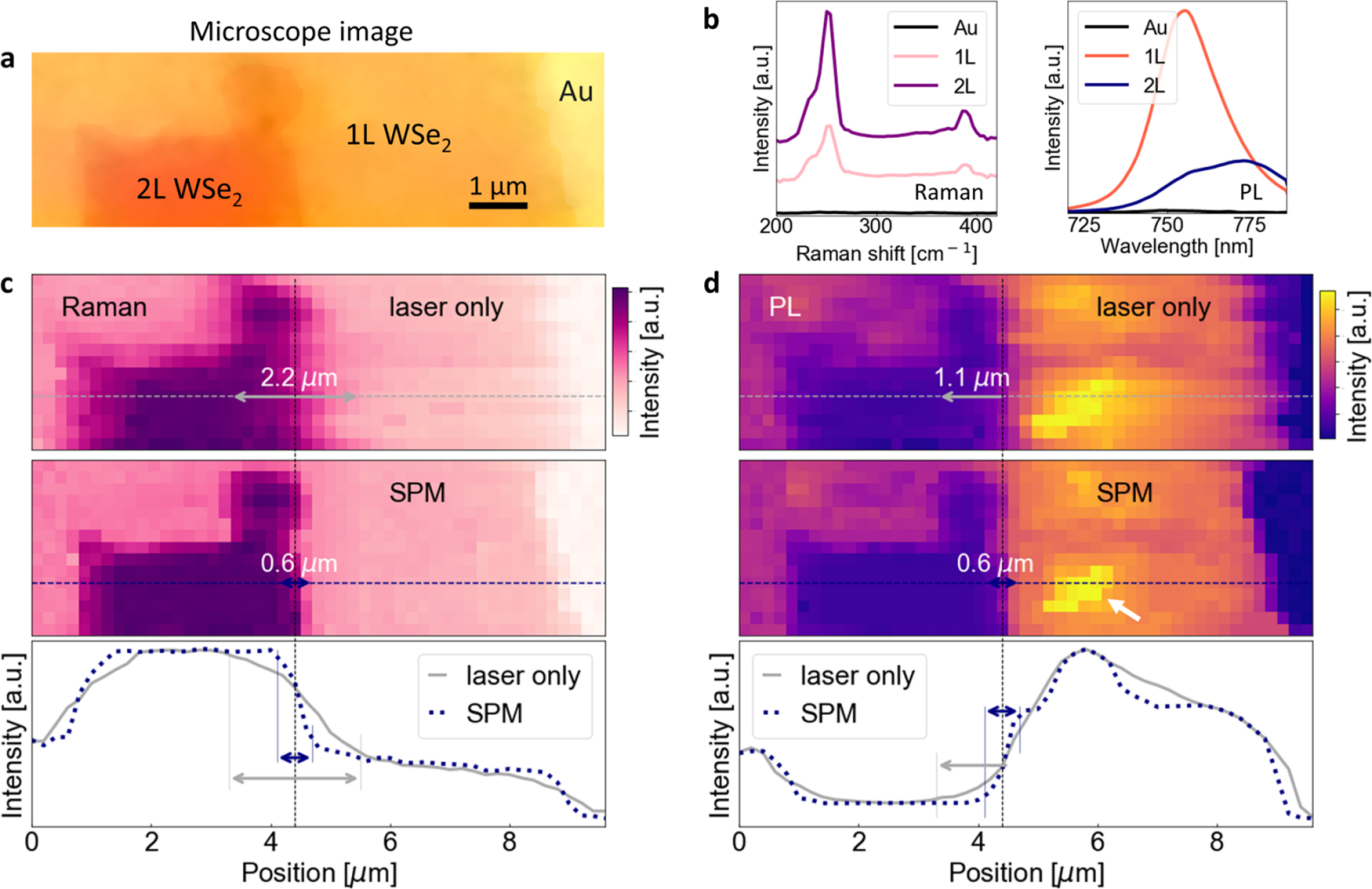
Enhancing the spectral resolution with SPEM. (a) Microscope image of the mapped WSe_2_ sample. (b) Raman (left) and PL (right) spectra produced by one and two layers of WSe_2_. Intensity maps of Raman (c) and PL (d) measured without (top) and with (middle) SPEM followed by intensity cross-section (bottom). SPEM improves spectral resolution from ∼2.2 μm to ∼600 nm.

A competitive resolution is achieved by the plasmonic enhancement provided by SPEM (Figure 2g) compared with conventional laser mapping. As a test sample, we chose WSe_2_, as presented in Figure 3a. To substantiate the accuracy of SPEM in detecting a single-molecular TMD layer, we illustrate the Raman and photoluminescence responses emitted by monolayer and bilayer WSe_2_, respectively, as depicted in Figure 3b. A comparison of intensity maps for standard laser mapping (top) and SPEM (bottom) is shown for Raman and PL (Figure 3c,d, respectively). SPEM impressively reduces spectral resolution from 2.2 μm (approximately equivalent to a laser spot size) to 600 nm, as highlighted in the intensity cross sections in Figure 3c,d. The resolution value is dictated by the stage in the setup and is not limited by the technique. The translation motion of the stage in the *x*–*y* plane has a minimum incremental motion of 500 nm along with a guaranteed bidirectional repeatability of ±1.25 μm. After every individual measurement, the stage advances ∼500 nm to position the samples at the next spot under the excitation laser or white light, hence limiting the achievable resolution. It is certainly possible to yield a higher resolution (ultimate achievable resolution dictated by NP contact facet, here 20–30 nm) with a sophisticated commercially available stage with significantly lower minimum incremental motion. SPEM’s high resolution allows precise identification of local structural variations sufficient to capture an observable difference in optical and electrical properties. This allows for mapping of morphological, optical, and electrical properties, thus providing three simultaneous data points, unlike the methods mentioned in Table 1.

As an example, in Figure 3d, the white arrow indicates a nanoscale enhancement in PL, likely due to local water absorption.^75^ Advances in nanotechnology demand the capacity to detect nanoscale cell-to-cell variability, preventing the commercialization of memristive switches,^76^ similarly for TMDs susceptible to doping.^3^

### 2.5 Local Nanoscale Morphological Tracking

Finally, SPEM represents a pivotal achievement in advancing the NPoM geometry.^50,55^ In the traditional NPoM method, AuNPs are randomly drop-cast on a test material. Unavoidable variability in AuNP geometries induces gap modes with differing wavelengths and intensities.^56,73^ Consequently, an extensive statistical analysis of the DF data from hundreds of AuNPs is required to uncover the true characteristics of the tested material.^73^ Furthermore, in NPoM, it is impossible to discern local variations at the nanoscale because of random AuNPs geometries and the necessity to maintain a distance of >1 μm between them to prevent hybridization of their modes.^65^ By attaching a single AuNP to a portable cantilever, SPEM allows for precise nanoscale DF mapping with a consistent AuNP, eliminating the need for extensive statistical analysis and enabling access to specific regions.

**Figure 4:**
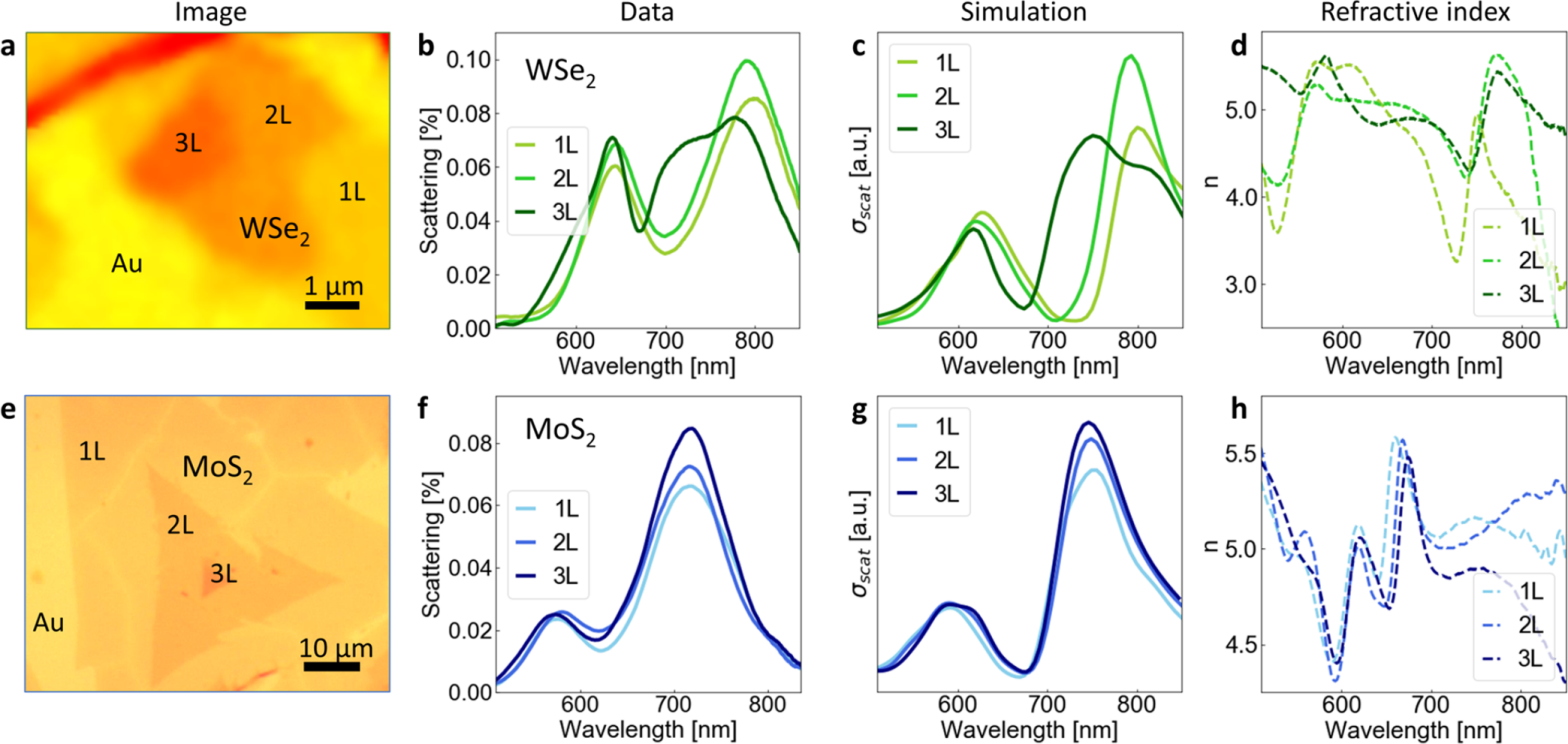
Plasmonic resonances in MoS_2_ and WSe_2_ (1–3 layers). Microscope pictures of examined WSe_2_ (a) and MoS_2_ (e) flakes. DF data for 1–3 layers of WSe_2_ (b) and MoS_2_ (f) match FDTD simulations (c, g, respectively). Pronounced changes in WSe_2_ modes for different thicknesses correspond to noticeable variations in refractive index (d), which are less significant for MoS_2_ (h). Data for (d) and (h) are taken from ref (52).

Figure 4 presents the capability of SPEM for selective nanoscale DF tracking. We individually placed SPEM nanoprobes on 1–3 layers of microsized WSe_2_ and MoS_2_ flakes (Figure 4a,4e, respectively). The used nanoprobe produces resonances at different energies compared to Figure 2d, as we employ thinner parylene coating to boost the intensity of the gap mode at ∼720 nm (see Figure S2b). The collected DF scattering data (Figure 4b,4f) closely align with finite-difference time-domain (FDTD) simulations presented in Figure 4c,g for WSe_2_ and MoS_2_, respectively, utilizing refractive indices (*n*) sourced from the literature (Figure 4d,4h).^52^

Interestingly, for WSe_2_, a significant disparity in the DF spectra is observed between 1 and 3 layers (Figure 4b), indicative of a distinct *n* (Figure 4d).^52^ Conversely, such pronounced variation is absent for 1–3 layers of MoS_2_ (Figure 4f), mirroring the similarity in *n* across a range of MoS_2_ thicknesses (Figure 4h).^52^ This confirms our ability to access precise nanosheet regions and discern local variations, a capability that cannot be achieved with the traditional NPoM setup.

### 2.6 Stability, Reproducibility, and Adaptability

The nanoprobes exhibit commendable stability and durability. They withstand abrupt contact with the sample’s surface (immediate downward step of 0.5 mm) and maintain integrity during horizontal movement in contact with a surface without needing a tapping mode (see Figure S1). The probe may become contaminated by airborne dust or pick up dirt from the sample (see Figure S1d); however, such contamination has not impacted the measurements to a considerable extent (Figure S5) unless the contaminants happen to land precisely on the AuNP, which is a rare occurrence.

The soft nature of the Au facets and the parylene coating ensure that the tested materials are not scratched or damaged. The applied pressure of approximately 0.037 GPa,^58^ with a force constant of 0.1 N/m^77^ does not negatively impact the optoelectrical properties of the tested material, as confirmed by the comparison of PL/Raman signals with and without SPEM (Figure 3).

SPEM nanoprobes demonstrate reproducibility, with consistent plasmonics across multiple probes and diverse materials (Figure S5). Finally, they are adaptable to any XYZR motion stage and microscope objective with a working distance ≥2.5 mm without necessitating ultrahigh vacuum or laser beam control for cantilever deflection as required for techniques listed in Table 1.

### 2.7 Outlook

This work presents only a few example materials (Figure S5). Still, SPEM can serve as electrical contacts for any thin-film material beyond MoS_2_ and WSe_2_, with spectroscopy enhancement generally more potent as excitons in most materials polarize in the direction of the gap resonance.^59^ For instance, in Figure S3, we present the DF data acquired with SPEM on a 20 nm thick polymer (sufficient to sustain field enhancement in the nanogap^64,65^), which enhances Raman intensity 20,000 times. Furthermore, the versatility of nanoprobes ought to extend to biological samples, thanks to the nonreactive and nonsharp characteristics of the Au electrode. Still, it should be noted that the optical enhancement declines significantly for materials thicker than 20 nm.^64^

Moreover, SPEM holds promise for mapping magnetic domains, as the DF relies on the material’s resistivity, which, in turn, is influenced by the magnetization direction, with the benefit of Raman spectra being sensitive to magnetic domains.^78^

The facet (contact area of AuNP and substrate) can be further reduced beyond the radius of 15 nm using smaller AuNPs (with diameters <80 nm). Still, variations in AuNP dimensions change optical enhancement’s intensity and wavelength.^59^ While horizontal electrical contact can be achieved by positioning two gold-coated cantilevers on either side of the test material without AuNP or a conductive substrate, this configuration does not provide enhanced spectroscopy or nanosized electrical pads.

Damage to the cantilever primarily results from overheating the parylene insulating layer due to excessive laser power or bias (see Figure S1d). This can be mitigated by setting current compliance to ≤10 nA and the laser power to ≤5 μW. Nevertheless, we advocate for research to investigate alternative coatings with *n* ≤ 1.6, focusing on deposition methods that do not jeopardize the integrity of microsized cantilevers. We note that we have already verified that cantilevers composed of silicon nitride (SiN) and indium tin oxide (ITO) are unsuitable for our purposes due to their luminescent interference (Figure S6) and susceptibility to breakage, respectively.

The setup step determines the resolution value, not the approach. After each measurement, the stage moves 500 nm to place the samples beneath the excitation laser or white light, limiting the resolution. A sophisticated commercial stage with a much lower minimum incremental motion will yield a higher resolution (the final attainable resolution, which is defined by the NP contact aspect, in this instance, 20–30 nm).

To simplify the fabrication of SPEM probes, a nanoparticle printer may be used to accurately deposit AuNPs onto the cantilever, rather than manually picking AuNPs on a specific substrate. Implementing this approach will not only enhance the throughput of preparing SPEM probes but also enhance the precision in positioning AuNP on the conducting cantilever.

## 3 Conclusions

We have introduced an innovative simultaneous optoelectrical characterization technique and demonstrated its capabilities on MoS_2_ and WSe_2_ nanosheets. SPEM combines the NPoM geometry with a portable conductive cantilever, allowing for the simultaneous acquisition of topographical, electrical, and spectroscopical data. At the same time, other techniques are limited to only one or two such characterizations. Our approach excels among scanning probe techniques in combining multiple simultaneous characterizations, ease of use, tip durability, and reproducibility. Using a nanoelectrode composed of AuNP, we achieve one of the smallest (*r* ≈ 15 nm) and cleanest vdW contacts that do not harm tested materials. Thanks to the local plasmonic enhancement, we accomplish a spectral resolution of 0.6 μm for TMDs, which is even higher for materials beyond TMDs. In summary, we believe that SPEM provides an exceptional tool for advancing nanomaterials.

## 4 Methods

### 4.1 Sample Preparation

The CVD MoS_2_ is synthesized on SiO_2_/Si utilizing elemental sulfur and MoO_3_ powder as precursors. During the growth, 2.5 mg of MoO_3_ and SiO_2_/Si substrate are set at 720 °C. 60 mg portion of sulfur at the upper stream is around 250 °C and 60 sccm of N_2_ is used as carrier gas. 0.5 mg/mL NaOH solution is used as a promoter, which is spin-coated on SiO_2_/Si. Similarly, CVD WSe_2_ is synthesized with elemental selenium and WO_3_ powder. During the growth, 10 mg of WO_3_ and SiO_2_/Si substrate is set at 750 °C. 50 mg of selenium at the upper stream is around 250 °C, and 20 sccm of forming gas (95% N_2_, 5% H_2_) is used as carrier gas. 15 mg/mL Na_2_WO_4_·2H_2_O solution is used as a promoter, which is spin-coated on SiO_2_/Si. The MoO_3_/WO_3_ is distributed in an alumina boat, in which a layer of molecular sieve is applied to control the precursor’s sublimation rate.

The surface-energy-assisted process^79^ is applied to transfer the CVD samples onto gold substrates. A piece of UV-Ozone-treated PDMS stamp (Gel-Film by Gel-Pak) is placed onto the surface of the growth substrate and gently pressed to ensure adequate adhesion between the PDMS stamp and CVD flakes. Subsequently, the edges of the substrate were immersed in DI water and slowly detach the PDMS stamp with CVD flakes from the substrate. They are then baked on a hot plate at 80 °C for 30 min to remove moisture. Finally, the CVD flakes are transferred onto Au through a deterministic viscoelastic stamping method.^46^

### 4.2 Nanoprobes Fabrication

We thermally evaporate a 6 nm Au/3 nm Cr thin conductive layer onto a transparent tipless 750 nm quartz-like cantilever^80^ to make SPEM nanoprobes. Using SmartAct XYZR piezoelectric positioners, we picked up a single AuNP from a clean insulating substrate. To enable electrical contact with a single AuNP, parylene is thermally deposited and etched to ≈60 nm to reveal its conductive facet.

### 4.3 Experimental Setup

A tungsten probe tip from Lambda Photometrics Ltd., connected to a custom-made XYZ manual positioner from Thorlabs, enables electrical contact with the underlying Au substrate. Electrical signals are transmitted from the top and bottom electrodes through triaxial cables to the Keithley 2634B source meter, facilitating low-noise measurements down to 10 pA.

Optical assessments are conducted by using a tailored optical setup. Spectra are acquired with integration times ranging from 0.5 to 2 s, employing a 100× 0.8-NA objective from an Olympus. A 633 nm C.W. single-longitudinal-mode laser from Integrated Optics is utilized for exciting PL/Raman signals, which are subsequently detected by a Kymera spectrometer linked to an Oxford Instruments Newton EMCCD camera. The laser power applied to the samples is 1.5 μW. DF signals are induced using white light bulbs (12 V 100 W GY6.35, Osram) and gathered through an optical fiber to an Ocean Optics spectrometer.

### 4.4 FDTD Simulations

We employ commercial Lumerical software.^81^ Throughout all configurations, we incorporate a layout validated in ref (55) consisting of AuNP (Ø = 80 nm) featuring a facet (Ø = 30 nm) encircled by a 1 nm layer of citrate (*n* = 1.4) with 1 nm of transfer residues below TMDs (*n* = 1.4).^82^ The intricate refractive indices are sourced from ref (52) for WSe_2_ and MoS_2_, from ref (83) for Au, and are established at *n* = 1.6 for parylene.^35^ Light is introduced at a 20° angle relative to the Au substrate to replicate the employed microscope setup.

## Supporting Information Available

Discussions on SPEM nanoprobes, FDTD method to study the tuning of plasmonic resonance, optical enhancement in SPEM probes, reproducibility of dark-field data using SPEM probes, and electronic band diagrams of MoS_2_ junction (PDF)
