# Supplementary material for: Scanning Plasmon-Enhanced Microscopy for Simultaneous Optoelectrical Characterization
Source: ACS Nano. 2024 Jul 27;18(31):20412–21. doi: 10.1021/acsnano.4c04671 (PMC11308916; doi:10.1021/acsnano.4c04671)
Supplement: Supplementary file 1 — nn4c04671_si_001.pdf [file nn4c04671_si_001.pdf]

Supplementary Information

Scanning Plasmon-Enhanced Microscopy for Simultaneous Opto-Electrical Characterization

Joanna Symonowicz†1, Atif Jan†1, Han Yan[1], Manish Chhowalla[1], Giuliana Di Martino[1*]

[1]University of Cambridge, Department of Materials Science and Metallurgy, 27 Charles Babbage Rd, Cambridge CB3 0FS, United Kingdom. e-mail: gd392@cam.ac.uk

†equal contribution

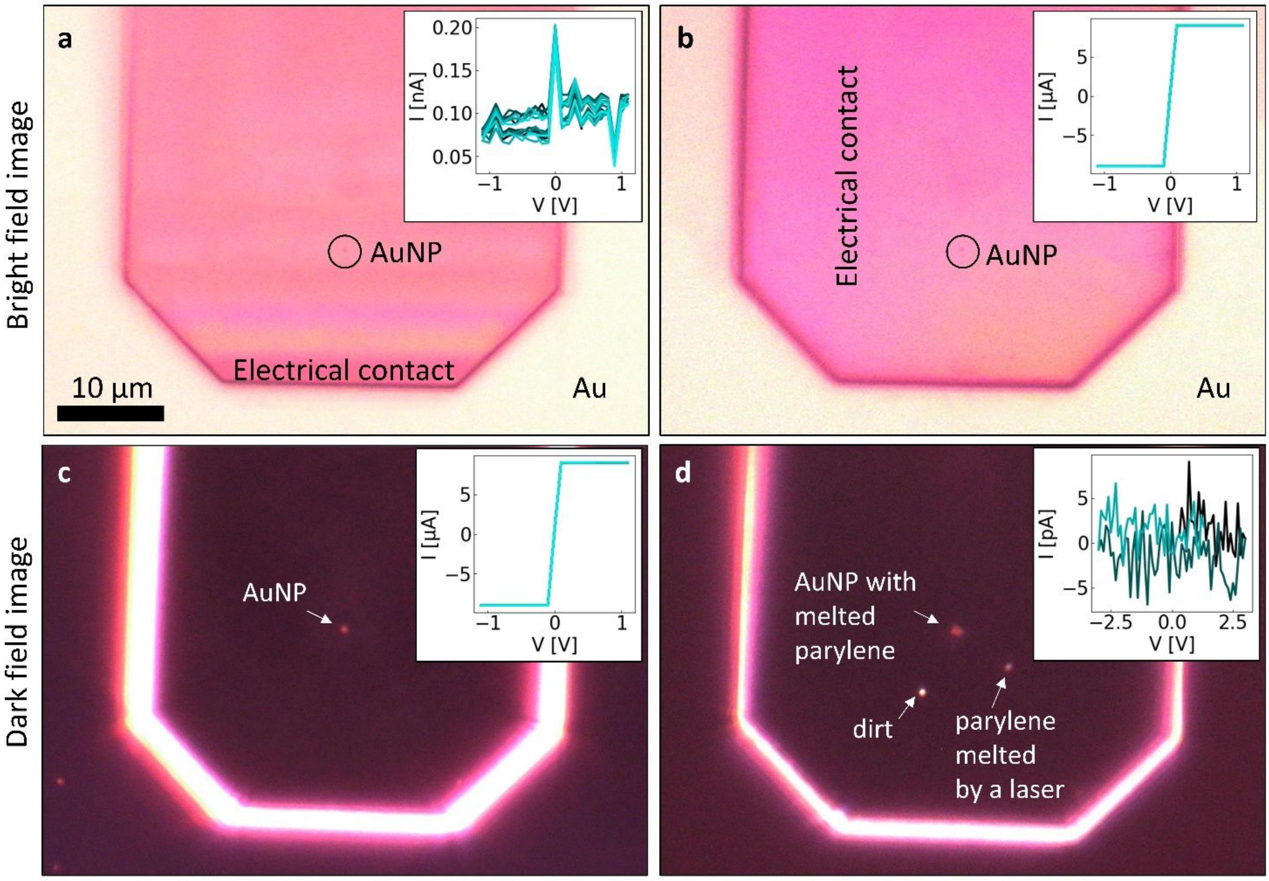

Fig. S1 |Bright (top) and dark (bottom) field microscope images of the SPEM nanoprobes. Current-voltage (*I*-*V*) characteristics achieved by contacting cantilever without (**a**) and through (**b**) a gold nanoparticle (AuNP). The *I*-*V* data confirms that an electrical contact is provided through AuNP exclusively and an insulating parylene layer prevents leakage voltages. (**c**) A dark field image of a clean functioning probe placed on Au substrate. (**d**) Nanoprobe breakdown – Joule heating coming from optical and electrical measurements melts parylene around AuNP and breaks an electrical contact. Dirt that has settled from the air is visible but does not disturb probe usage. A melted parylene spot is induced by the exposure of 444 nm laser power of 7 μW for 15 s. Safe laser power for the cantilever is established at 5 μW.

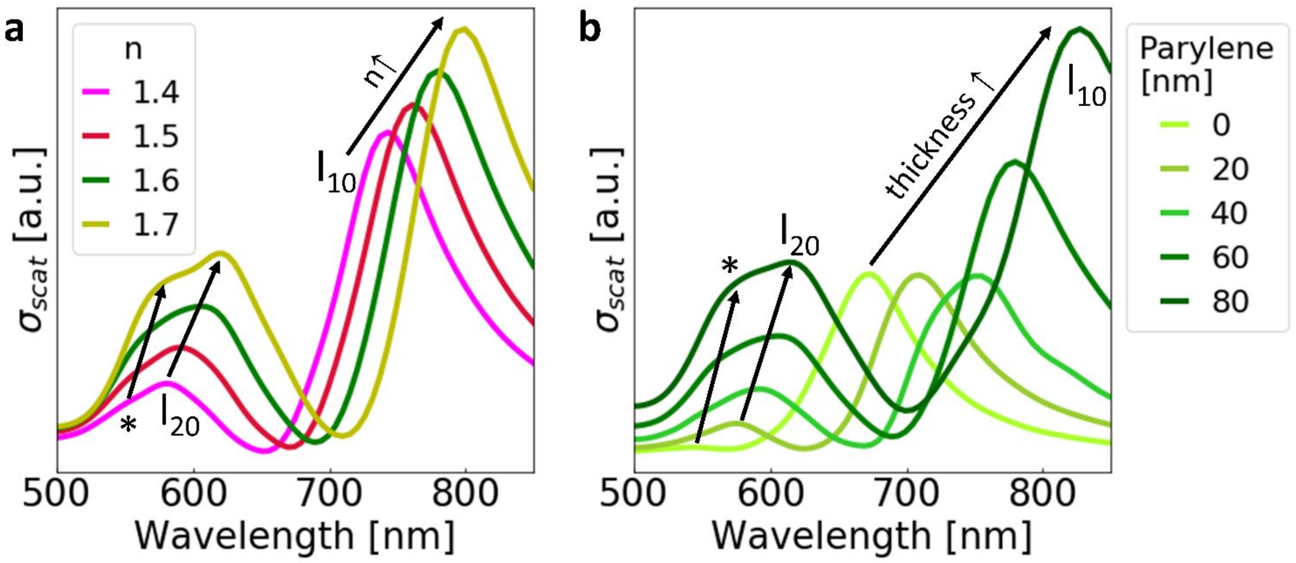

Fig. S2 | Simulation using the Finite-Difference Time-Domain (FDTD) method to study the tuning of plasmonic resonance through polymer coating. (**a**) Influence of the refractive index (*n*) with polymer thickness of 60 nm. SPEM employs parylene (n ≈ 1.6) whereas NPoM uses PMMA (n ≈ 1.5), which explains the blueshift of SPEM modes in Fig. 2d. The intensity of simulated peaks mismatch experiment shown in Fig. 2d due to the limitations of the used objective (LMPLFLN100X from Olympus) which is designed for wavelengths of 400-700 nm with transmission dropping to 17% at 900 nm. (**b**) Modulating parylene thickness from 0 to 80 nm results in a redshift of modes, particularly l_{10}, and a significant amplification of the single (marked by *) and l_{20} modes.

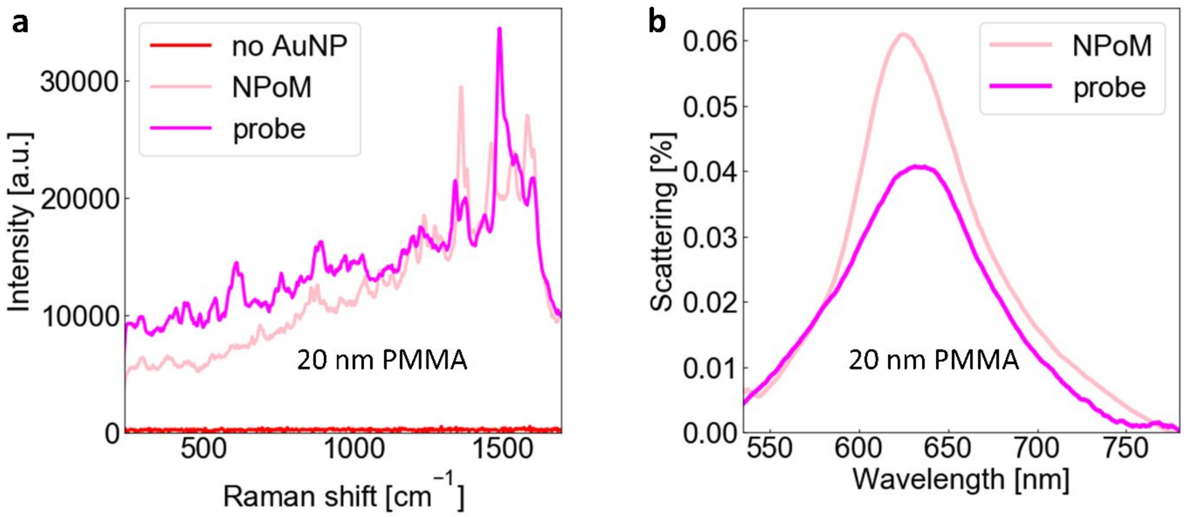

Fig. S3 | Optical enhancement with SPEM nanoprobes. (**a**) A significantly higher Raman spectra enhancement of ~30,000 times for PMMA compared to transition metal dichalcogenides (TMDs) discussed in the main paper (~2 times). This difference is attributed to the orientation of excitons in TMDs, which is transverse to the plasmonic resonances introduced by the nanoprobes. (**b**) Comparison of dark field (DF) spectra between a standard nanoparticle on mirror (NPoM) geometry and SPEM (probe), showing very similar optical results.

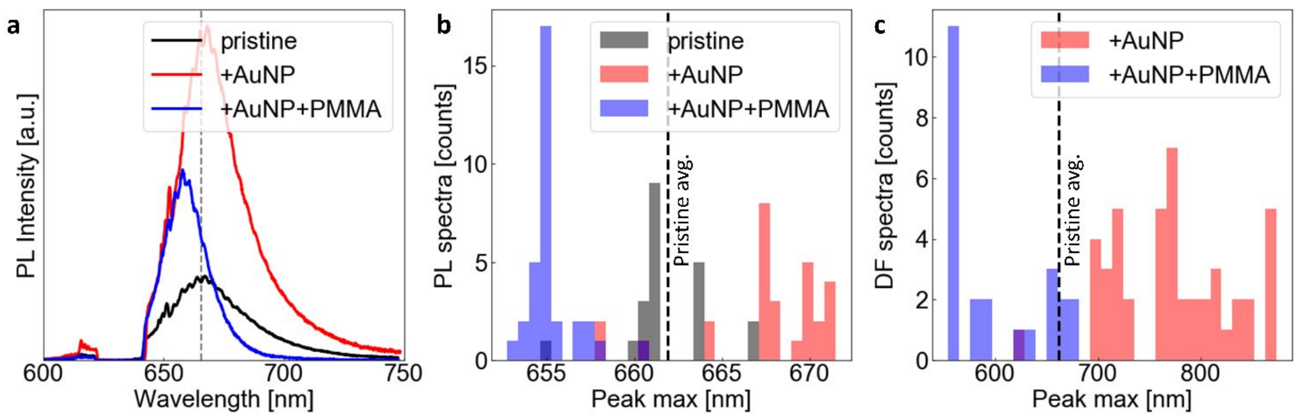

Fig. S4 | Correlation between MoS_2 photoluminescence (PL) and plasmonic enhancement wavelengths (λ). AuNP induces maximum plasmonic enhancement at λ > PL, while additional PMMA amplifies plasmons oscillating at λ < PL, resulting in apparent red- and blueshifts in PL, respectively. (**a**) PL spectra of a selected MoS_2 flake (black line) with AuNP (red line) and with AuNP + PMMA (blue line). (**b**) Statistical analysis of λ corresponding to maximum peak intensities for pristine and plasmon-enhanced PL of MoS_2. The dashed line indicates the average PL peak position of pristine MoS_2. (**c**) Statistical analysis of plasmonic peak maxima for the AuNP/MoS_2/Au structure both without (red) and with (blue) PMMA.

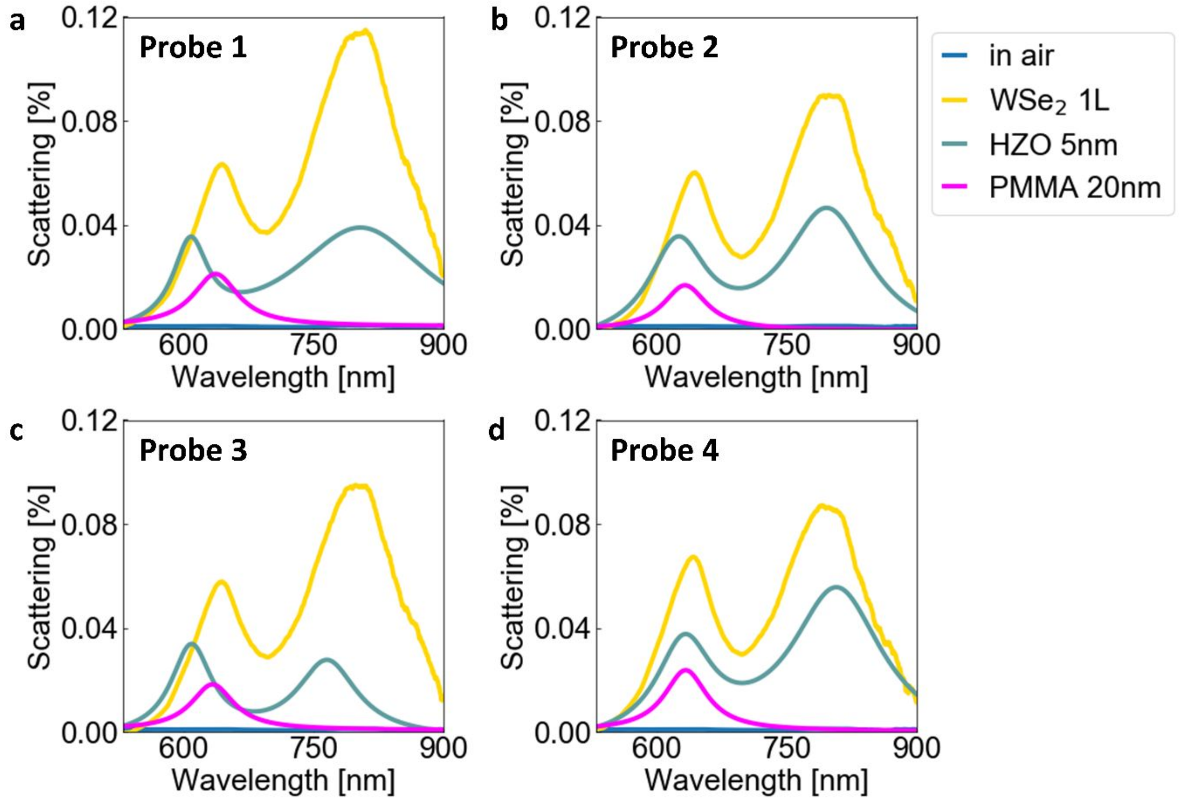

Fig. S5 | Reproducibility of the spectral DF response for four nanoprobes. The position of a plasmonic gap mode depends on materials' *n* and thickness. Slight differences are due to local variations in the properties of tested materials and shape of AuNP on a probe.

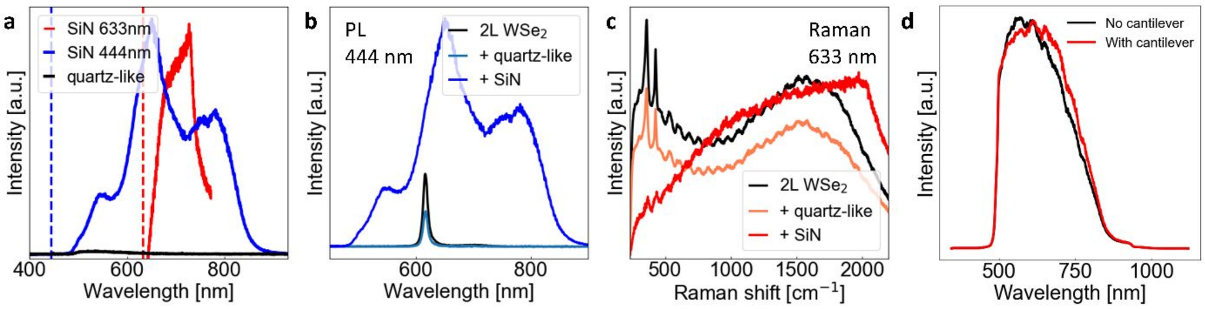

Fig. S6 | Comparison between standard SiN and 'quartz-like' cantilevers used in this work. (**a**) Luminescent spectra produced by the cantilevers suspended in air for 444 nm (blue) and 633 nm (red) excitation wavelengths. 'Quartz-like' cantilevers are not luminescent regardless of excitation energy. Comparison of PL (**b**) and Raman (**c**) spectra produced by two layers of WSe_2 collected via cantilevers of different materials. Spectra of the SiN probe entirely overshadows the signals from WSe_2, making it unsuitable for our applications. (d) Reference spectra produced by white light in quartz like cantilever compared with no cantilever.

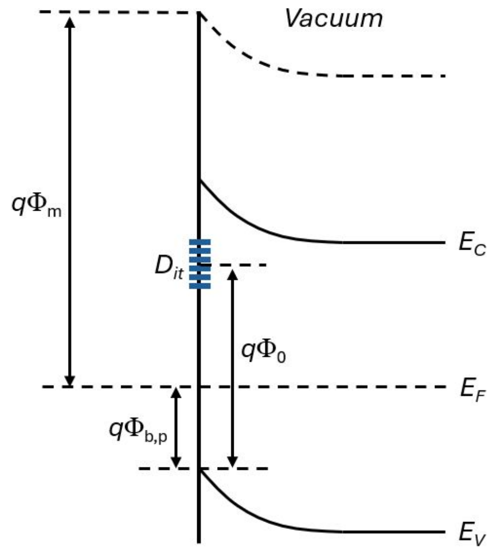

Fig. S7 | *Band diagram of Au-bulk MoS_2 junction*. *The symbols are: E_C, energy level of MoS_2's conduction band; E_F, Fermi level after thermal equilibrium; E_V, energy level of MoS_2's valance band; Φ_m, Au work function; Φ_0, neutral level of interface defect states; $\Phi_{b,p}$, Schottky barrier height for holes; D_{it}, interface defect states density. E_C, E_F, E_V, $q\Phi_m$, $q\Phi_0$, $q\Phi_{b,p}$ all have unit eV. D_{it} has unit states cm^{-2} eV^{-1}. The band alignment of Au and bulk MoS_2 with a pristine van der Waals contact (i.e. no significant interface defect state density D_{it}). A clean metal/semiconductor junction between MoS_2 (valance band energy $E_V \sim 5.75$ eV) and Au (work function $q\Phi_m \sim 5.30$ eV) results in a small Schottky barrier height for holes ($q\Phi_{b,p} = E_V - q\Phi_m$, $\sim$ 0.45 eV). Therefore, holes can transfer from Au to MoS_2 more easily than electrons, which gives p-type I-V behaviour. However, many studies demonstrate that metal-MoS_2 junctions always show n-type I-V behaviour despite the work functions of metals (Nat Rev Phys 4, 101–112, 2022; ACS Nano 11, 2, 1588–1596, 2017). This phenomenon is attributed to Fermi level pinning effect at the metal-semiconductor interfaces, such as chemical reactions and damages introduced during metal deposition. When the interface defect state density is large ($D_{it} \rightarrow \infty$), the Fermi level at the interface will be pinned by the interface states ($E_F \sim E_V - q\Phi_0$), and the barrier height is independent of the metal work function. Since these interface defect states locate near MoS_2's conduction band, the Schottky barrier height for holes will become higher than that for electrons. Consequently, the I-V characteristics will be n-type. Based on this analysis, we conclude that the p-type behaviour in our measurements suggests a pristine contact between the Au electrodes and bulk MoS_2.*
